# Antileishmanial Activity of* Handroanthus serratifolius* (Vahl) S. Grose (Bignoniaceae)

**DOI:** 10.1155/2017/8074275

**Published:** 2017-02-12

**Authors:** Erica Vanessa Souza Costa, Heliton Patrick Cordovil Brígido, João Victor da Silva e Silva, Marlia Regina Coelho-Ferreira, Geraldo Célio Brandão, Maria Fâni Dolabela

**Affiliations:** ^1^Programa de Pós-Graduação em Ciências Farmacêuticas, ICS, Universidade Federal do Pará, Belém, PA, Brazil; ^2^Departamento de Botânica, Museu Paraense Emílio Goeldi, Belém, PA, Brazil; ^3^Escola de Farmácia, Universidade Federal de Ouro Preto, Ouro Preto, MG, Brazil

## Abstract

This study aimed to evaluate the leishmanicidal activity of ethanol extract, fractions, and isolated substance from* Handroanthus serratifolius* against* Leishmania amazonensis*. Furthermore, this activity was related to cytotoxicity, and the selectivity index was determined. The ethanol extract was obtained by maceration of the stem powder, and the extract was subjected to fractionation on chromatographic column. The lapachol was obtained by acid base extraction followed by purification in chromatographic column. The antipromastigote activity and cytotoxicity tests were carried out by the cell viability method (MTT). Modified THP-1 cells were infected with* L. amazonensis* promastigotes and treated for 24 h with different concentrations of the extract, fractions, and lapachol. The ethanol extract, dichloromethane, and ethyl acetate fractions were not active against promastigotes (IC_50_ > 200 *μ*g/mL) or cytotoxic (CC_50_ > 500 *μ*g/mL), and the selectivity index (SI) was greater than 2.5. The ethyl acetate fraction was active only in promastigotes; it is not cytotoxic (CC_50_ > 500 *μ*g/mL, SI > 5). The lapachol was selectively active only against amastigote (IS > 2.5, CC_50_ > 500 *μ*g/mL). In summary, lapachol and ethyl acetate fraction are promising against amastigote and promastigote forms, respectively.

## 1. Introduction

Leishmaniasis is caused by over 20* Leishmania* species and it is transmitted to humans by the infected phlebotomine female sandflies. There are three main forms of the disease: visceral leishmaniasis (VL), cutaneous leishmaniasis (CL), and mucocutaneous leishmaniasis. It is estimated that about 200,000 to 400,000 new cases of VL occur worldwide each year. Over 90% of new cases occur in 6 countries: Bangladesh, Brazil, Ethiopia, India, South Sudan, and Sudan. The cutaneous leishmaniasis is the most common form of leishmaniasis. About 95% of CL cases occur in the Americas, the Mediterranean basin, the Middle East, and Central Asia. Almost 90% of mucocutaneous leishmaniasis cases occur in Bolivia, Brazil, and Peru [1].

The leishmaniasis control is based on vector combat, extermination of infected dogs, and treatment of infected individuals [2]. The amphotericin and* N*-methyl glucamine antimoniate (Glucantime®) [3] are drugs currently used in the treatment of leishmaniasis. However, they have problems as severe adverse effects, and some strains have already presented increased parasitic resistance [2, 4, 5]. In addition, all drugs are currently available for parenteral administration [4]. As a result, many patients abandon treatment; this fact favors the appearance of resistant strains [6].

In this context, plant species are the best and greatest source of drugs for mankind. Ethnobotanical studies have demonstrated the popular use of plants in the treatment of leishmaniasis both orally and in the topical application on lesions [7, 8]. Many plants present in their composition substances of the classes of alkaloids, terpenes, naphthoquinones, lignans, chalcones, flavonoids, and sesquiterpene lactones, compounds described in the literature as effective in leishmanicidal activity [9–11].

The search for alternative therapies for leishmaniasis is very important. Many species of the Bignoniaceae family are used in folk medicine to treat external ulcers, skin diseases, and skin disorders [7]. However, the antileishmanial activity of these species has not been tested yet.

**Figure 1:**
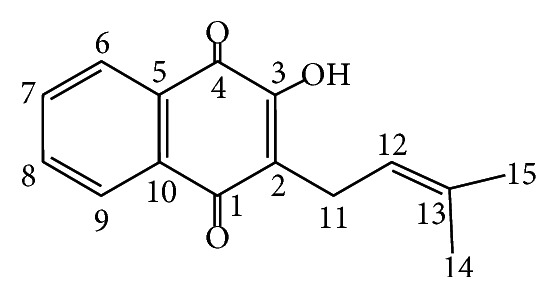
Chemical structure lapachol.

*Handroanthus serratifolius* (Bignoniaceae) is used in traditional medicine as antitumor, antiparasitic, and antimalarial agent [12–14]. Originally the following substances were isolated from species of the Bignoniaceae family as* Handroanthus serratifolius*, (Figure 1), *α*-1,4-naphthoquinone-methylfuran, dehydro-*α*-lapachone, *α*-lapachone, tecomaquinone I, and dehydroiso-*α*-lapachone [15].

The antipromastigote activity of lapachol, isolapachol, and dihydrolapachol, with soluble derivatives (potassium salt), was evaluated. All substances inhibited the growth of* Leishmania amazonensis* and* L. brasiliensis* promastigotes, with a greater effect in* L. amazonensis*. The lapachol showed activity in* L. amazonensis* (IC_50_ = 5.2 *μ*g/mL) than* L. braziliensis* (IC_50_ = 11.9 *μ*g/mL) [16].

Other studies evaluated the leishmanicidal activity of lapachol and compared its efficacy with a reference drug, sodium stibogluconate (Pentostam®). These compounds were evaluated against amastigotes of* Leishmania* (*Viannia*)* braziliensis* (LVb). In vitro, lapachol exhibited antiamastigote effect, whereas in vivo it did not prevent the development of LVb induced lesions at an oral dose of 300 mg/kg/day for 42 days. Pentostam® demonstrated a significant antiamastigote effect in vitro and in vivo (60 mg/kg/day). Perhaps the lapachol inhibits the microbicide function of macrophages in vivo. Alternatively, it might be transformed into an inactive metabolite(s) or neutralized, losing its leishmanicidal activity [17].

This study aimed to evaluate the leishmanicidal activity of ethanol extract, fractions, and isolated substance obtained from* Handroanthus serratifolius* against* Leishmania amazonensis*. Furthermore, this activity is related to cytotoxicity determining the selectivity index.

## 2. Material and Methods

### 2.1. Plant Material and Extraction

Plants were collected on 10 March 2014 in Emílio Goeldi Museum, Pará, Brazil (S 01°27′3.031′′, W 48°26′40.2′′). The voucher specimen (MG 206637) was deposited in the João Murça Pires Herbarium.

**Figure 2:**
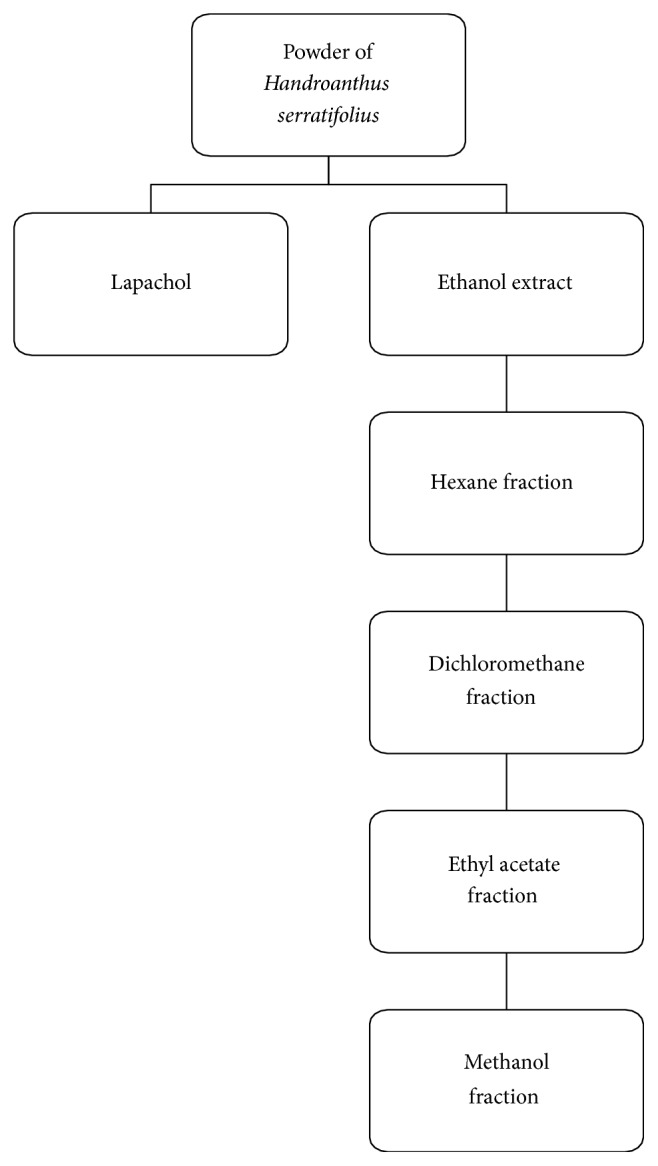
Extract, fractions, and isolated substance obtained of* Handroanthus serratifolius.*

Plants were dried at room temperature for seven days. The material was powdered and extracted with ethanol by cold maceration. The resultant solution was concentrated in a rotary evaporator to obtain the ethanol extract. The extract was fractioned in chromatographic column (CC) with silica gel as stationary phase and increasing polarity solvents (hexane, dichloromethane, ethyl acetate, and methanol) as mobile phase (Figure 2).

The powder of* H. serratifolius* was treated with 2.5% sodium carbonate solution for 24 h for lapachol isolation. The solution was filtered, and the precipitate was solubilized in aqueous hydrochloric acid. After 30 minutes, it was centrifuged (3,000 rpm/10 minutes) and a yellow solid precipitate was separated (Figure 2). The precipitate was dried and submitted to fractionation on chromatographic column. Nuclear magnetic resonance was used to identify the isolated compounds.

*Lapachol.* NMR ^1^H (200 Hz, CDCl_3_): 8,13 (dd, *J* = 6.2 and 1.4 Hz); 8,05 (dd, *J* = 7.6 and 1.4 Hz); 7,78 (dt, *J* = 6.2 and 1.4 Hz); 7,63 (dt, *J* = 7.3 and 1.4 Hz); 7,34 (dt, *J* = 7.3 and 1.4 Hz); 5,21 (m); 3,30 (d, *J* = 7,3 Hz); 1,79 (s); 1,68 (s). NMR ^13^C (50 Hz, CDCl_3_): 184,4 (C-4); 181,71 (C-1); 152,72 (C-2); 134,77 (C-7); 133,73 (C-13); 133,04 (C-8); 132,78 (C- 5); 129,54 (C-10); 126,78 (C-6); 126,02 (C-9); 123,57 (C-3); 119,73 (C-12); 25,67 (C-14); 22,65 (C-11); and 17,85 (C-15).

### 2.2. Antileishmanial Activity of* Leishmania amazonensis*

#### 2.2.1. Antipromastigotes Assay

Strains isolated from leishmaniasis (*Leishmania amazonensis* MHOM/BR/2009/M26361) were obtained from the Evandro Chagas Institute, Ananindeua, Brazil.

The* L. amazonensis* promastigotes were cultivated at 26°C in RPMI 1640 medium supplemented with 10% heat-inactivated fetal bovine serum (Gibco®, Grand Island, NY, USA), penicillin (100 U/mL), and streptomycin (100 *μ*g/mL) [18].

Culture of promastigote forms in logarithm phase was adjusted to 5 × 10^6^ parasites/100 *μ*L. The susceptibility testing was performed in 96-well plates. The extract, fraction, and lapachol were tested in triplicate in a concentration gradient (200 to 3.125 *μ*g/mL). Negative control was performed with parasites and incubation medium. The positive control was made with amphotericin B (25–0.3906 *μ*g/mL). After 24 h of incubation at 26°C in 5% de CO_2_, 10 *μ*L of tetrazolium salt (5 mg/mL) was added to each well, and the parasites were quantified in enzyme-linked immunosorbent-assay plate reader. The IC_50_ was determined by linear regression (Graph Pad Prism version 5.04). The results were classified as follows: IC_50_ ≤ 100 *μ*g/mL were considered active, IC_50_ between 101 and 200 *μ*g/mL were considered moderate active, and IC_50_ ≥ 200 *μ*g/mL were considered to be inactive [18].

#### 2.2.2. Antiamastigote Assay

Modified THP-1 cell (4 × 10^5^ cells/0.1 mL) was cultured in RPMI-1640 (Roswell Park Memorial Institute 1640) medium (Sigma Aldrich®, USA), supplemented with 5% of fetal calf serum, kept in a 5% CO_2_ atmosphere at 37°C with phorbol ester as inducing agent. The cells were added the circular coverslips (2 × 10^5^); then* L. amazonensis* promastigotes were added (5 × 10^6^). The samples treatment was performed with concentrations of 250, 125, and 62,5 *μ*g/mL/24 h. The coverslips were removed and stained with Giemsa. After that, the infection rate of macrophages was determined.

### 2.3. Viability Assay and Selective Index

Cell viability was determined by the MTT [3-(4,5-dimethylthiazol-2-yl)-2,5-diphenyl tetrazolium bromide] [19]. Modified THP-1 cell (4 × 10^5^ cells/0.1 mL) was cultured in RPMI-1640 (Roswell Park Memorial Institute 1640) medium (Sigma Aldrich®, USA), supplemented with 5% of fetal calf serum, kept in a 5% CO_2_ atmosphere at 37°C. The cells were treated with extracts, fractions, or lapachol in different concentrations (between 500 and 25 *μ*g/mL). MTT was added (5.0 mg/mL) after 24 h of further incubation. The plate was incubated at 37 C in an atmosphere of 5% CO_2_ for 4 h. Dimethyl sulfoxide was added to each well to solubilize the formazan crystals. The optical density was determined at 490 nm (Stat Fax 2100 microplate reader, Awareness Technology, Inc., USA). The cell viability was expressed as percentage of the control absorbance (absorbance of control group) in the untreated cells after subtracting the appropriate background. The cytotoxic concentration (CC_50_) was determined by linear regression. Samples with CC_50_ > 500 *μ*g/mL were considered of low cytotoxicity. Selectivity index (SI) for the antipromastigote activity was calculated based on the rate between CC_50_ and IC_50_ for the in vitro activity against* L. amazonensis* [20].

## 3. Results and Discussion

In this study, lapachol (C_15_H_14_O_3_) was isolated from stem powder of* H. serratifolius*. However, other studies isolated ethanol extract of* H. serratifolius* lapachol (2.9% yield) [21]. The antipromastigote activity of lapachol has been described in posterior study [16].

**Figure 3:**
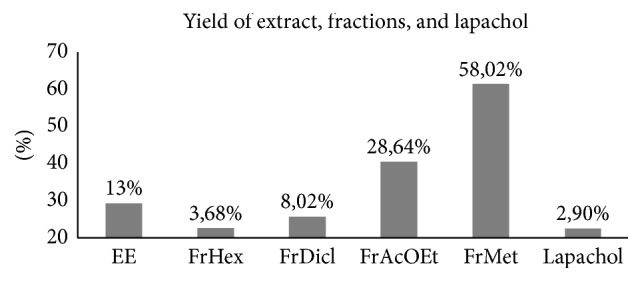
Yield of extract, fractions, and isolated substance obtained of* Handroanthus serratifolius*. EE: ethanol extract; FrHex: hexane fraction; FrDcl: dichloromethane fraction; FrAcOEt: ethyl acetate fraction; FrMet: methanol fraction.

To verify if* H. serratifolius* has other substances with antileishmanial activity, the ethanol extract of the stem was obtained (13% yield). This extract was fractioned resulting in four fractions: hexane (3.68% yield), dichloromethane (8.02% yield), ethyl acetate (28.64% yield), and methanol (58.02% yield) (Figure 3).

Lapachol, ethanol extract, and fractions were tested against* L. amazonensis* promastigotes. Unlike a previous study [16], lapachol was not active in* L. amazonensis* promastigotes (IC_50_ > 200 *μ*g/mL; Table 1). The* L. amazonensis* strain used in this assay was isolated from a patient who had previously not responded to conventional therapy. This may explain the divergent response.

The ethanol extract, dichloromethane, and methanol fractions did not show activity against promastigotes (IC_50_ > 200 *μ*g/mL; Table 1). The ethyl acetate fraction was promising (IC_50_ < 100 *μ*g/mL; Table 1). Study on thin-layer chromatography (results not shown) suggests coumarins in ethyl acetate fraction. Coumarins were isolated from* H. impetiginosa* [22].

The 7-{[(2R^*∗*^)-3,3-dimethoxyloxiran-2-2-yl]methylorixan}-8-[(2R^*∗*^,3R^*∗*^)-3-isopropenyloxira-2-yl]-2H-chromen-2-one, phebalosin, and 7-methoxy-8-8(4-methyl-3-3-furyl)-2H-chromen-2-one were tested against* Leishmania panamensis* amastigotes. The coumarins were active (IC_50_ 9.9, 10.5 and 14.1 mg/mL, resp.) and cytotoxic in human promonocytic U-937 cells (CC_50_ 9.7, 33.0 and 20.7, resp.; [23]). The fractionation of the ethyl acetate fraction may contribute to antipromastigote activity.

We assessed the cytotoxicity of all samples for modified THP-1 cell line. Extract, fractions, and lapachol showed no toxicity for this cell (CC_50_ > 500 *μ*g/mL; Table 1). Similarly, another study showed that the ethanol extracts of leaves and flowers from* H. aureus* were not cytotoxic for macrophages rats (CC_50_ > 1000 *μ*g/mL) [24]. Unlike this study, several studies describe the cytotoxicity of lapachol [25–27]. The most active fraction against promastigotes showed higher selectivity index (SI > 5). Lapachol showed selectivity index greater than 2.5 (Table 1).

**Figure 4:**
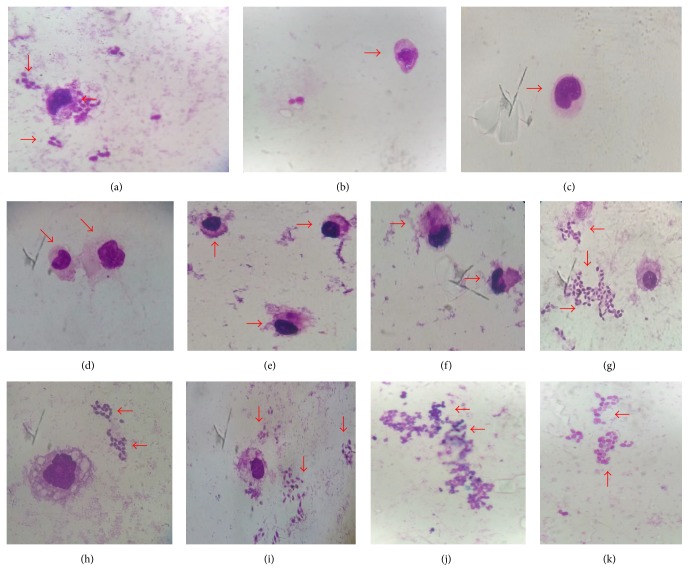
Antiamastigote activity of* Handroanthus serratifolius*. (a) Negative control; (b) macrophage without infection; (c) amphotericin B (50 *μ*g/mL); (d, e, and f) lapachol (250 *μ*g/mL, 125 *μ*g/mL, and 62.5, resp.); (g) ethanol extract; (h) hexane fraction: (i) dichloromethane fraction; (j) ethyl acetate fraction; and (k) methanol fraction (250 *μ*g/mL); increase of 100x.

Lapachol reduced the infection of macrophages, with greater effect observed at 250 *μ*g/mL (Figure 4; Table 2). Antiamastigote activity of lapachol against* Leishmania* (*Viannia*)* braziliensis* was described [17]. This effect has been linked to stabilization of the complex and DNA topoisomerase [28]. Some have antiparasitic effect as time-dependent [29, 30]. Thus, increased exposure time can contribute to the inhibitory effect.

## 4. Conclusion

The ethanol extract, hexane, dichloromethane, and methanol fractions from* H. serratifolius* showed no antipromastigote and antiamastigote activities. It was also not cytotoxic. The ethyl acetate fraction showed selective effect for promastigotes, while lapachol was active for amastigotes.

## Figures and Tables

**Table 1:** Antipromastigote activity and cytotoxicity of *Handroanthus serratifolius.*

Sample	*L. amazonensis*		
	Promastigote CI_50_ (*µ*g/mL)	CC_50_ (*µ*g/mL)	SI
EE	>200	>500	>2.5
FrHex	>200	>500	>2.5
FrDicl	>200	>500	>2.5
FrAcOEt	<100	>500	>5.0
FrMet	>200	>500	>2.5
Lapachol	>200	>500	>2.5
Amphotericin B	>0,390625	>100	256

IC_50_: inhibitory concentration 50%; CC_50_: concentration cytotoxic 50%; IS: selectivity index; EE: ethanol extract; FrHex: hexane fraction; FrDcl: dichloromethane fraction; FrAcOEt: ethyl acetate fraction; FrMet: methanol fraction.

**(a):** 

Sample	% reduction		
	concentration (*µ*g/mL)		
	250	125	62,5
EE	0	0	0
FrHex	0	0	0
FrDicl	0	0	0
FrAcOEt	0	0	0
FrMet	0	0	0
Lapachol	53.97	44.45	22.49

**(b):** 

Sample	% concentration (*µ*g/mL)		
	50	25	12,5
Amphotericin B	86.7	81.5	79.9

EE: ethanol extract; FrHex: hexane fraction; FrDcl: dichloromethane fraction; FrAcOEt: ethyl acetate fraction; FrMet: methanol fraction.
